# On Bandaging and Other Operations of Minor Surgery

**Published:** 1848

**Authors:** 


### ARTICLE III.

*On Bandaging and other Operations of Minor Surgery.* By F. W. SARGENT, M. D. Philadelphia: Lea & Blanchard. 1848. pp. 379. (From the publishers: for sale by Keen & Brother, Chicago.)

As a general rule, books which are intended to give but the mere outlines of a subject are objectionable, from the fact that they afford to students and others the means of gaining a passable amount of information upon the subject treated of, without labor or much mental effort, to the exclusion of better works and more laborious research.

Taking this view of the matter, we were not favorably impressed with the title of the work before us; but we are happy in being able to state that upon a more thorough examination of the book, we have become convinced that it contains much valuable information upon bandaging, instruments, apparatus, and the methods of performing minor operations, such as bleeding, arresting hemorrhage, closing wounds, introducing the ctaheter, &c., not to be found in more elaborate treatises, but which, nevertheless, is of vast importance to the young surgeon. H.

